# Clinical Utility of Modified Inflation–Deflation Test in Eustachian Tube Function Testing

**DOI:** 10.3390/diagnostics16162534

**Published:** 2026-08-11

**Authors:** Dong-Hoon Lee, Yun Heo, Han-Seul Na, Seokhwan Lee, Se-Joon Oh, Sung-Won Choi

**Affiliations:** 1 Department of Otorhinolaryngology and Biomedical Research Institute, Pusan National University Hospital, Busan 49241, Republic of Korea; ehdgns0627@naver.com (D.-H.L.); nahanseul@gmail.com (H.-S.N.); entlsh85@gmail.com (S.L.); o3jdoc@hanmail.net (S.-J.O.); 2 Department of Otorhinolaryngology, Gyeongsang National University Changwon Hospital, Changwon 51472, Republic of Korea; whdgnsa07@naver.com; 3 Department of Otorhinolaryngology-Head and Neck Surgery, College of Medicine, Pusan National University, Busan 50612, Republic of Korea

**Keywords:** eustachian tube, modified inflation–deflation test, eustachian tube dysfunction, tympanic membrane perforation, sonotubometry

## Abstract

**Background/Objectives:** The modified inflation–deflation test (MIDT) is a physiological test used to evaluate eustachian tube function in patients with tympanic membrane perforation. However, the clinical characteristics and diagnostic value of MIDT have not been fully established. This study aimed to investigate the characteristics of MIDT findings in normal function and patients with Eustachian tube dysfunction (ETD) classified by sonotubometry. **Methods:** We retrospectively assessed 192 ears of 181 patients with tympanic membrane perforation who underwent sonotubometry and MIDT. Based on sonotubometry findings, 117 ears were classified into the normal function group and 75 ears into the ETD group. The MIDT parameters of the groups, including the first opening pressure (OP1), first closing pressure (CP1), residual pressure after three openings (RP3), and pressure difference between OP1 and CP1 were compared. Correlation and receiver-operating characteristic (ROC) analyses were performed. **Results:** The ETD group had significantly higher OP1, CP1, and RP3 levels than the normal function group (all *p* < 0.001). The pressure difference between OP1 and CP1 was significantly greater for the normal function group. Failure of Eustachian tube opening was observed in 46.7% and 3.4% of ears in the ETD and normal function groups, respectively. Eustachian tube opening duration measured by sonotubometry was negatively correlated with OP1, CP1, and RP3 but positively correlated with the OP1–CP1 difference. ROC analysis identified an optimal cutoff value of 112.50 daPa for the OP1–CP1 difference, yielding a sensitivity of 92.3% and a specificity of 58.7%. **Conclusions:** MIDT was significantly associated with sonotubometry findings and effectively differentiated ETD from normal Eustachian tube function. These findings support the clinical utility of the MIDT as a physiological tool for assessing Eustachian tube function in patients with tympanic membrane perforation.

## 1. Introduction

The Eustachian tube is an S-shaped canal connecting the middle ear cavity to the nasopharynx. Its primary functions are to ventilate the middle ear, equalize middle ear pressure with atmospheric pressure, protect the middle ear from nasopharyngeal secretions, and facilitate mucociliary clearance through transient opening during swallowing or yawning. Eustachian tube dysfunction (ETD) occurs when these functions are impaired, resulting in ineffective pressure equalization, reduced middle ear ventilation, and impaired clearance of middle ear secretions. Persistent ETD may cause symptoms such as aural fullness, conductive hearing loss, tinnitus, and otalgia, and may contribute to middle ear diseases including tympanic membrane retraction, otitis media with effusion, chronic otitis media, and cholesteatoma, ultimately reducing quality of life [1,2,3,4,5,6,7,8].

Methods for evaluating Eustachian tube function can be broadly classified into conventional, radiological, and physiological approaches. Conventional methods, such as the Valsalva and Toynbee maneuvers, can only confirm Eustachian tube opening and do not provide quantitative assessment. Radiological techniques, including temporal bone computed tomography performed during the Valsalva maneuver, visualize the anatomical structure of the Eustachian tube but cannot evaluate its physiological function. Therefore, physiological tests are the primary methods used in clinical practice because they allow quantitative functional assessment as well as confirmation of tube opening [9,10,11,12,13,14,15,16,17].

Among the physiologic tests for evaluating Eustachian tube function, Bluestone’s nine-step test and the simplified Williams method using a 226 Hz probe tone are the most commonly used. Both methods assess tympanic membrane mobility by comparing tympanometric peak pressure before and after swallowing under positive and negative pressure conditions. Because these tests require an identifiable tympanometric peak, they cannot be performed in ears with a type B tympanogram, including those with otitis media with effusion or tympanic membrane perforation [18,19].

Physiological tests that can be performed regardless of tympanic membrane perforation include sonotubometry, tubotympano-aerodynamic graphy (TTAG), and the modified inflation–deflation test (MIDT). Sonotubometry evaluates active Eustachian tube opening by detecting changes in the intensity of a sound delivered through the nasal cavity at the external auditory canal during swallowing, allowing quantitative assessment of Eustachian tube opening duration and ventilatory function [20,21]. In contrast, the TTAG evaluates passive Eustachian tube function by measuring tympanic membrane movement in response to middle ear pressure changes induced by maneuvers such as the Valsalva maneuver, deep inspiration, and the Toynbee maneuver while the nose is occluded, thereby determining whether passive tubal opening and closure occur. Despite these advantages, both tests are performed via the nasal cavity and are therefore susceptible to the influence of sinonasal disease, which may affect the accuracy and reproducibility of the test results. In contrast, the MIDT is performed using only a probe inserted into the external auditory canal and does not require access through the nasal cavity. Positive or negative air pressure is applied directly to the middle ear via the external auditory canal, allowing Eustachian tube function to be evaluated in ears with tympanic membrane perforation. The original inflation–deflation test evaluates Eustachian tube function by applying positive or negative middle-ear pressure through a tympanic membrane perforation and primarily records the pressure at which the Eustachian tube opens during swallowing. In contrast, the modified inflation–deflation test (MIDT) uses an automated tympanometric system to continuously monitor middle-ear pressure during repeated swallowing, enabling assessment of both passive Eustachian tube opening in response to externally applied pressure and active Eustachian tube opening through pressure equalization. Unlike the original test, MIDT also records both opening and closing pressures and captures dynamic pressure changes throughout repeated swallowing, providing a more comprehensive evaluation of Eustachian tube function. Consequently, because the test does not rely on the nasal cavity, it is relatively less affected by sinonasal conditions. The modified inflation–deflation test (MIDT) is not a newly developed technique but refers to the inflation–deflation approach that has been used clinically for several decades to assess Eustachian tube function. Therefore, MIDT may serve as a useful physiological test for evaluating Eustachian tube function, particularly in patients with tympanic membrane perforation [10,22,23,24].

However, MIDT currently lacks well-established diagnostic criteria, and few studies have investigated the characteristic findings of MIDT for other Eustachian tube disorders. Therefore, this study evaluated the diagnostic performance of MIDT by comparing its results with sonotubometry in adults with chronic tympanic membrane perforation.

## 2. Materials and Methods

### 2.1. Participants

This study included 1330 ears of 665 patients who underwent Eustachian tube function testing at the Department of Otolaryngology of Pusan National University Hospital between December 2024 and November 2025.

Patients with chronic tympanic membrane perforation secondary to chronic otitis media were initially identified through otoendoscopic examinations performed by an otolaryngologist. Among these patients, those who underwent both MIDT and sonotubometry as part of their clinical evaluation were selected for further analysis. Sonotubometry was performed using the JK-05A (Rion, Kokubunji, Tokyo, Japan), and participants were classified into groups according to the sonotubometry findings.

**Figure 1:**
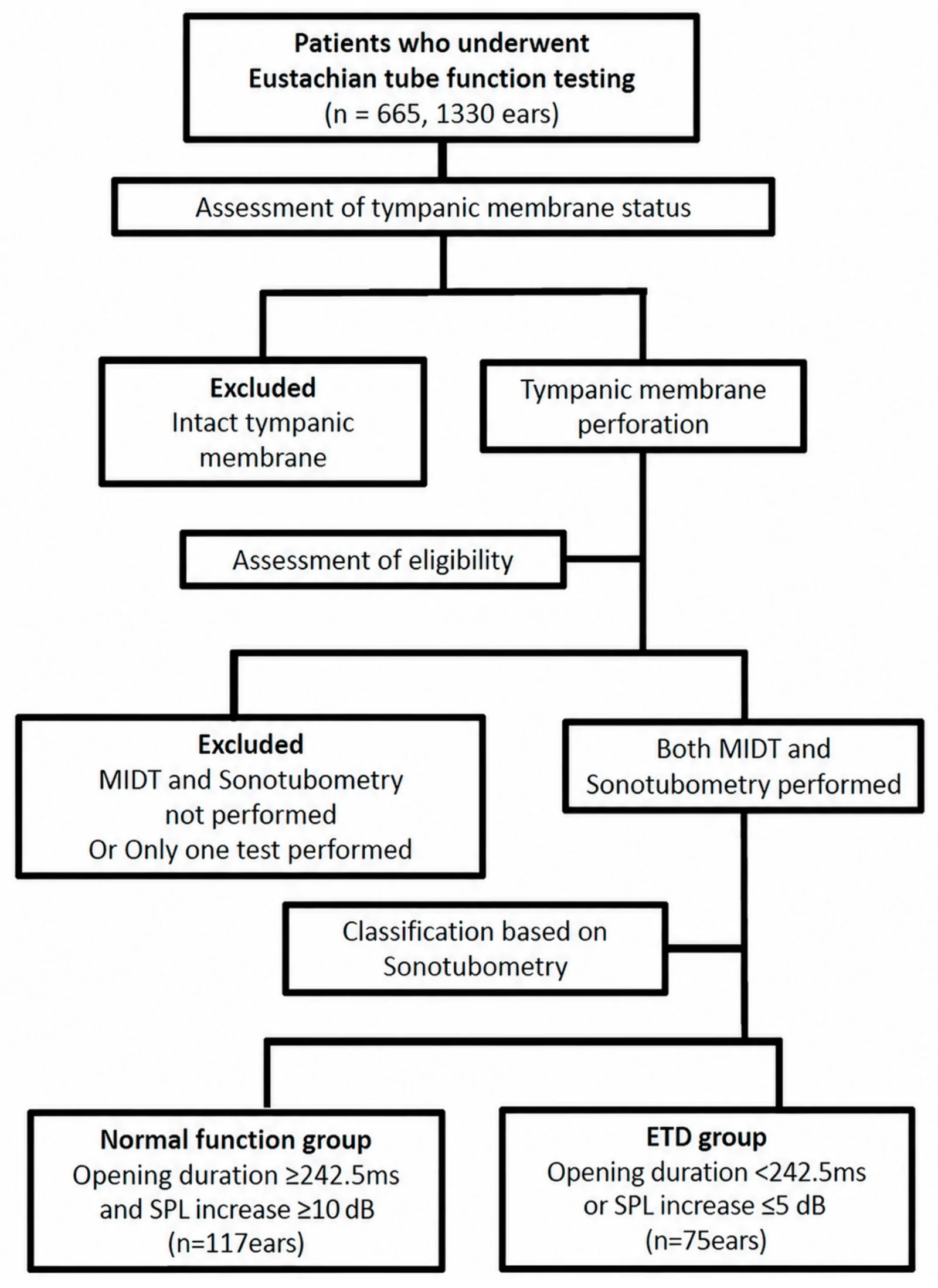
Flow diagram of participant selection and group classification.

Patients with patulous Eustachian tube were excluded because of the marked variability in symptoms and Eustachian tube functional patterns, as well as the limited number of cases, which could compromise the consistency of statistical analyses. In addition, patulous Eustachian tube has pathophysiologic characteristics distinct from the primary objective of the present study, which focused on evaluating the opening and closing function of the Eustachian tube. Accordingly, participants showing a stimulus sound pressure level below 100 dB or demonstrating an ‘open plateau pattern’ were excluded. Ultimately, the data of 192 ears from 181 participants were included in the final analysis. The sonotubometry-based classification criteria were adopted from Lee et al. (2024) [25]. Eustachian tube opening duration was automatically displayed as a numerical value by the system. In contrast, sound pressure change was not provided as a quantitative measurement but was visually assessed based on the waveform relative to the built-in 5 dB and 10 dB reference lines. Ears with an opening duration ≥ 242.5 ms and a waveform reaching or exceeding the 10 dB reference line were classified as normal, whereas ears with an opening duration < 242.5 ms or a waveform at or below the 5 dB reference line were classified as ETD. Ears with waveform amplitudes between the 5 dB and 10 dB reference lines were excluded to avoid ambiguous classification. Because the instrument does not provide a numerical value for sound pressure change, waveform amplitude was classified according to the built-in 5 dB and 10 dB reference lines displayed by the system (Figure 1) [25].

### 2.2. Study Procedures

All study procedures were approved by the Institutional Review Board (IRB No. 2601-021-159, date: 29 January 2026). Among the participants with tympanic membrane perforation, sonotubometry was first performed to group the participants. Subsequently, the classified participants underwent the MIDT using the GSI TympStar Pro (Grason-Stadler, Eden Prairie, MN, USA).

**Figure 2:**
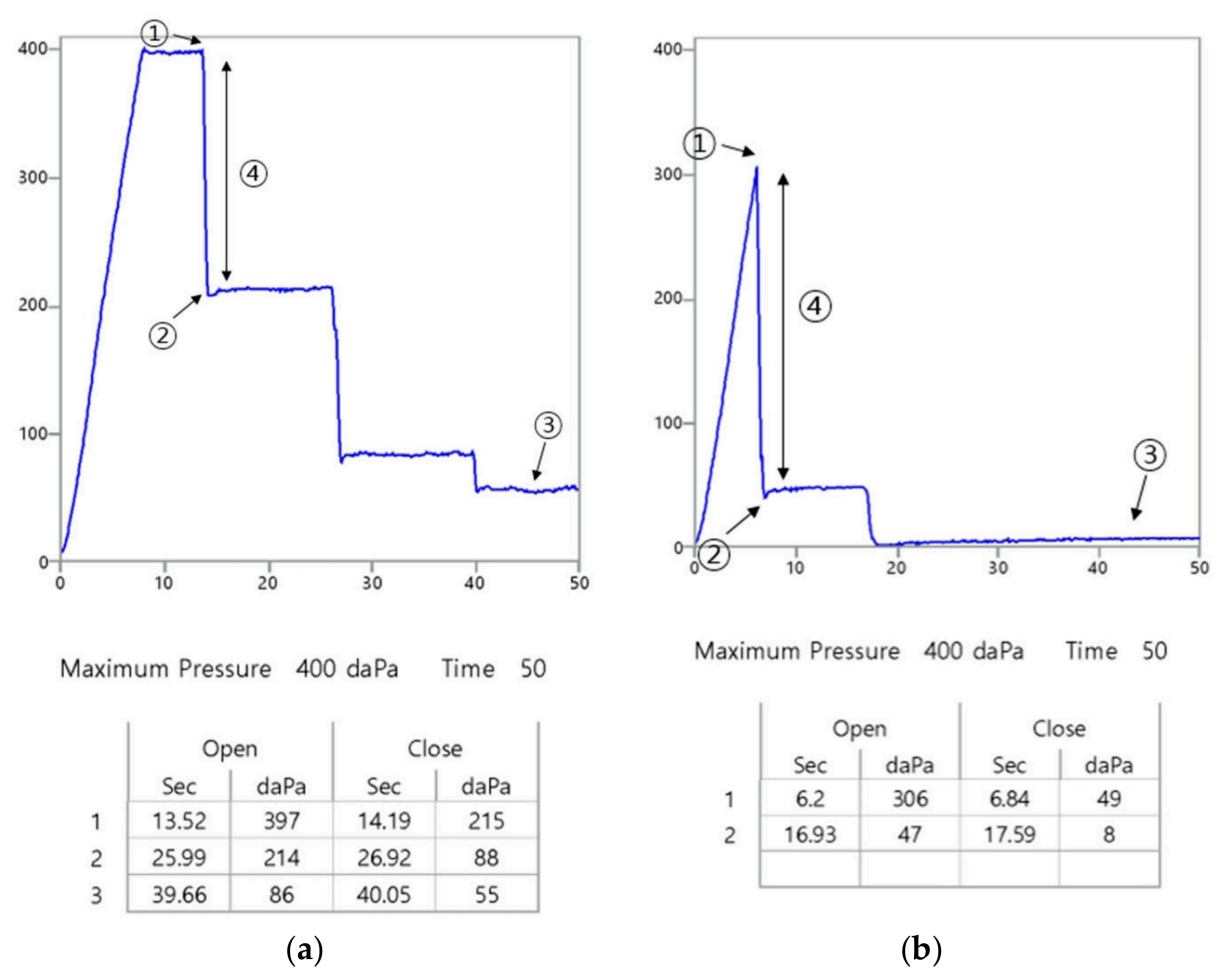
Representative examples of MIDT measurements. (**a**) Representative case showing no passive Eustachian tube opening up to 400 daPa pressurization, with only an active opening induced by swallowing. (**b**) Representative case of passive Eustachian tube opening during pressurization. ①, first opening pressure (OP1); ②, first closing pressure after initial opening (CP1); ③, residual pressure after three openings (RP3); ④, pressure difference between OP1 and CP1.

All MIDT measurements were performed using the inflation mode only, as negative pressure measurements may be influenced by middle ear effusion and middle ear structural abnormalities. A maximum inflation pressure of 400 daPa, which is the maximum pressure output of the instrument, was applied for all measurements. During the 50 s measurement period predetermined by the instrument, participants were instructed to perform at least three dry swallows (Figure 1). Most participants completed three swallows within the measurement period, whereas more than three swallows were uncommon. To ensure standardized measurements across all participants, only parameters derived from the first three swallowing events were analyzed. The following parameters were collected for statistical analysis: first opening pressure (OP1; ① in Figure 2), first closing pressure after initial opening (CP1; ② in Figure 2), and residual pressure after three openings (RP3; ③ in Figure 2). When no Eustachian tube opening was detected during the measurement period, the value was recorded as 400 daPa, corresponding to the maximum measurable pressure of the instrument. These ears were retained in the analysis because the absence of detectable opening represents a clinically relevant finding indicating that the Eustachian tube did not open within the measurable pressure range. However, these values were considered right-censored at 400 daPa because the actual pressure required for opening could not be determined.

### 2.3. Statistical Analysis

All statistical analyses were performed using SPSS version 28 (IBM Corp., Armonk, NY, USA). Differences in MIDT parameters between the groups were assessed using the Mann–Whitney U test. Spearman rank correlation analysis was performed to evaluate associations between variables, and receiver operating characteristic (ROC) curve analysis was conducted to evaluate the discriminative performance of MIDT parameters. For ROC analysis, higher values of OP1, CP1, and RP3 and lower values of OP1–CP1 were defined as the positive direction for ETD.

## 3. Results

### 3.1. Patient Characteristics

A total of 192 ears were included in the final analysis and were classified into the normal function and ETD groups according to the sonotubometry findings. The normal function group comprised 117 ears, including 41 male and 76 female ears, with a mean age of 59.85 ± 12.74 years. The ETD group comprised 75 ears, including 20 male and 55 female ears, with a mean age of 56.89 ± 14.41 years. Comparisons of the baseline demographic characteristics demonstrated no statistically significant differences in either sex distribution or mean age between the two groups, indicating that the groups were comparable with respect to these variables (Table 1). Among the 75 ears classified as ETD, 53 (70.7%) met both the opening duration and sound pressure change criteria, 18 (24.0%) met only the opening duration criterion, and 3 (4.0%) met only the sound pressure change criterion.

### 3.2. Comparison of MIDT Parameters

Comparison of the MIDT parameters demonstrated significant differences between the normal function and ETD groups across all evaluated variables. The first opening pressure (OP1) was significantly lower in the normal function group (344.78 ± 58.03 daPa) than in the ETD group (380.15 ± 34.63 daPa), indicating that a lower pressure was required to achieve the initial opening of the Eustachian tube in the normal function group. Similarly, the first closing pressure after the initial opening (CP1) was significantly lower in the normal function group (107.85 ± 94.24 daPa) than in the ETD group (258.40 ± 152.82 daPa). Furthermore, the pressure difference between OP1 and CP1 (④ in Figure 1), representing the magnitude of pressure equilibration following the initial opening, was significantly greater in the normal function group (236.93 ± 87.71 daPa) than in the ETD group (121.75 ± 133.61 daPa). The residual middle ear pressure after three repeated dry swallowing maneuvers (RP3) was also significantly lower in the normal function group (58.73 ± 82.33 daPa) than in the ETD group (237.19 ± 162.75 daPa), demonstrating a greater reduction in residual pressure following repeated swallowing in the normal function group (Table 2).

### 3.3. Eustachian Tube Opening Failure

**Figure 3:**
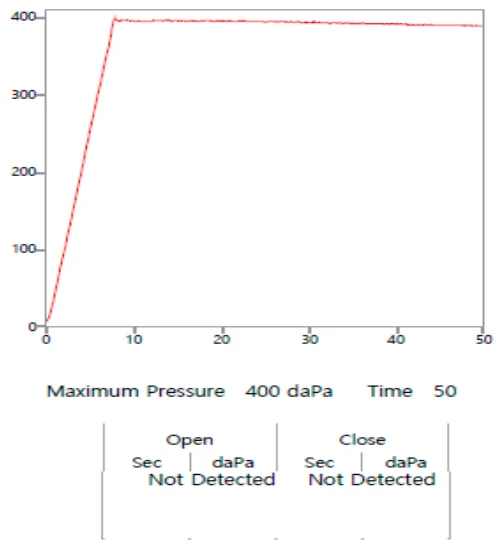
No detectable Eustachian tube opening during pressure loading or swallowing.

Failure of Eustachian tube opening during the MIDT was observed considerably more frequently in the ETD group than in the normal function group. Specifically, 4 of the 117 ears (3.4%) in the normal function group and 35 of the 75 ears (46.7%) in the ETD group failed to demonstrate Eustachian tube opening despite the application of positive pressure loading or repeated dry swallowing maneuvers. The difference in the frequency of Eustachian tube opening failure between the two groups was statistically significant (*p* < 0.001), indicating that unsuccessful tube opening during the MIDT was substantially more common in ears with ETD than in normal function ears (Table 3, Figure 3).

### 3.4. Correlation Analysis

Correlation analysis between the MIDT parameters and Eustachian tube opening duration measured by sonotubometry demonstrated significant associations for all evaluated parameters. Eustachian tube opening duration showed significant negative correlations with the first opening pressure (OP1; *r* = −445, *p* < 0.001), the first closing pressure after the initial opening (CP1; *r* = −0.521, *p* < 0.001), and the residual middle ear pressure after three repeated swallowing maneuvers (RP3; *r* = −0.602, *p* < 0.001). Among these variables, RP3 demonstrated the strongest negative correlation with Eustachian tube opening duration. In contrast, the pressure difference between OP1 and CP1, representing the magnitude of pressure equilibration after the initial opening, showed a significant positive correlation with Eustachian tube opening duration (*r* = 0.430, *p* < 0.001). Overall, all MIDT parameters were significantly correlated with Eustachian tube opening duration measured by sonotubometry (all *p* < 0.001) (Table 4).

### 3.5. ROC Analysis

**Figure 4:**
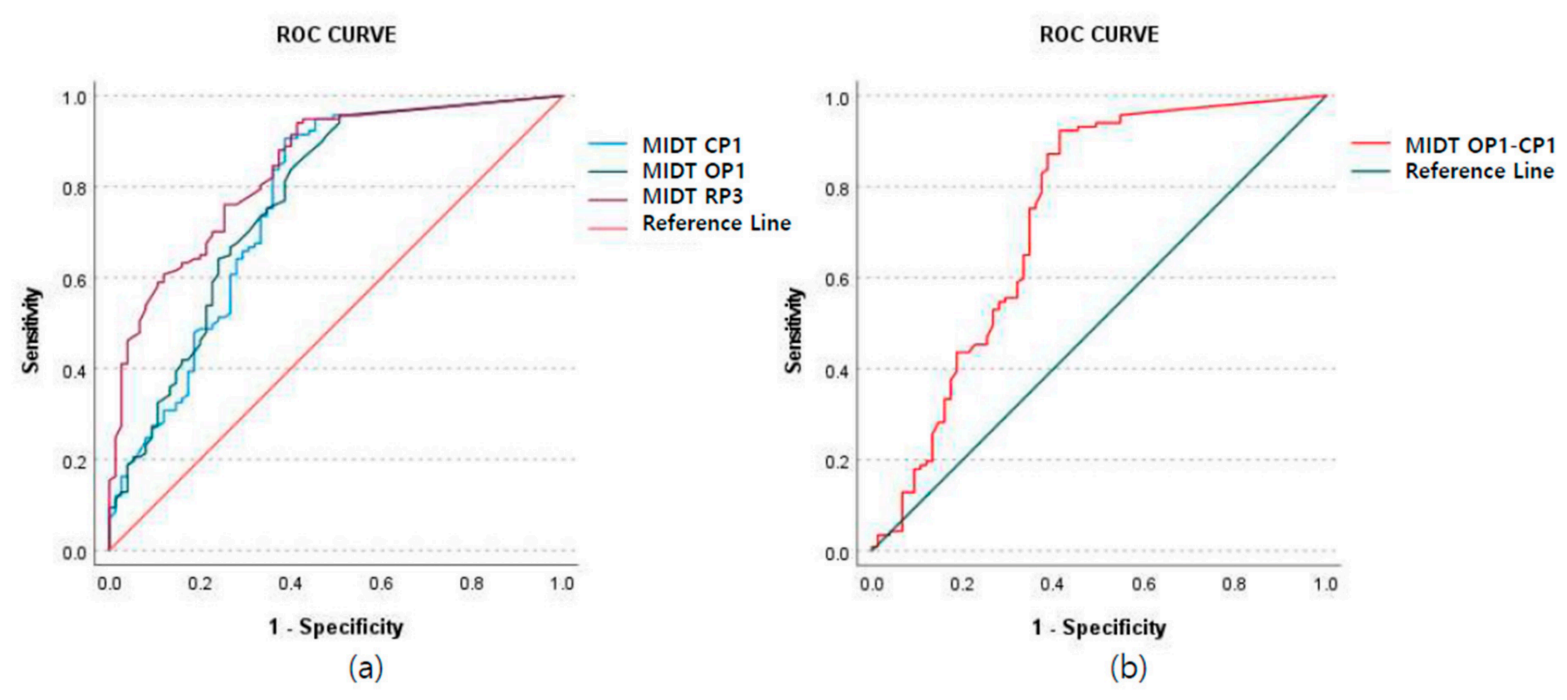
Receiver operating characteristic (ROC) curves for the MIDT parameters. (**a**) ROC curves for CP1, OP1, and RP3, for which higher values indicate abnormal sonotubometry findings. (**b**) ROC curve for OP1−CP1, for which lower values indicate abnormal sonotubometry findings. ROC, receiver operating characteristic; MIDT, modified inflation–deflation test.

ROC curve analysis was performed to evaluate the diagnostic performance of the MIDT parameters and to determine their optimal cutoff values for differentiating the ETD and normal function groups. Among the evaluated parameters, only the pressure difference between OP1 and CP1 demonstrated significant discriminatory performance. The optimal cutoff value, determined using the maximum Youden index (sensitivity + specificity − 1), was 112.50 daPa. At this threshold, the sensitivity and specificity for identifying ETD were 92.3% and 58.7%, respectively. These findings indicate that the pressure difference between OP1 and CP1 showed the best discriminative performance among the evaluated MIDT parameters for distinguishing ears classified as having ETD from those with normal Eustachian tube function (Table 5, Figure 4).

To assess the potential effect of including bilateral ears from the same patient, a sensitivity analysis was performed using only one ear per patient. For patients with bilateral data, only the right ear was included in the analysis. The results were consistent with those of the primary analysis, with no changes in statistical significance or the overall interpretation of the findings

## 4. Discussion

The present study classified ears into normal function and ETD groups according to sonotubometry findings and investigated the characteristics of MIDT in each group, thereby primarily evaluating the agreement between two physiological tests rather than the diagnostic accuracy of MIDT against a definitive clinical gold standard. The results demonstrated that the ETD group had significantly higher first opening pressure (OP1), first closing pressure after the initial opening (CP1), and residual middle ear pressure after three repeated swallowing maneuvers (RP3) than the normal function group. In contrast, the pressure difference between OP1 and CP1 was significantly greater in the normal function group. Furthermore, Eustachian tube opening failure during the MIDT occurred significantly more frequently in the ETD group, and all MIDT parameters showed significant correlations with Eustachian tube opening duration measured by sonotubometry. Among the evaluated parameters, the pressure difference between OP1 and CP1 demonstrated the greatest diagnostic performance for differentiating ETD from normal function ears.

Although significant differences were observed in the absolute values of the MIDT parameters between the two groups, the pressure difference between OP1 and CP1 was additionally analyzed to evaluate the magnitude of pressure change during the initial opening of the Eustachian tube. This pressure difference was significantly greater in the normal function group than in the ETD group, suggesting more effective pressure equilibration during the initial opening of the Eustachian tube in normal function ears. Furthermore, the proportion of ears in which neither passive Eustachian tube opening in response to positive pressure loading nor active opening during repeated swallowing was observed was significantly higher in the ETD group (46.7%) than in the normal function group (3.4%). Notably, the four ears in the normal function group without detectable opening on MIDT were classified as having normal Eustachian tube function by sonotubometry, the reference method. This finding highlights the imperfect concordance between physiological tests of Eustachian tube function and indicates that MIDT should be interpreted as a complementary assessment rather than a standalone diagnostic test.

These findings suggest that ears with ETD have a higher likelihood of failing to achieve both passive opening induced by positive pressure loading and active opening during swallowing throughout the MIDT. Furthermore, even when the Eustachian tube opened in response to pressure loading or swallowing, the pressure equilibration achieved with each opening event was limited, resulting in persistently higher residual middle ear pressure following repeated swallowing maneuvers.

During the MIDT, ears in which neither passive Eustachian tube opening induced by positive pressure loading nor active Eustachian tube opening during repeated swallowing was observed were significantly more common in the ETD group than in the normal function group. These findings are consistent with previous studies reporting that impaired Eustachian tube function is associated with a reduced likelihood of both passive and active Eustachian tube opening, as well as decreased ventilatory efficiency even when tube opening occurs [25,26].

Additionally, receiver operating characteristic (ROC) curve analysis was performed to identify the optimal cutoff values of the MIDT parameters for distinguishing the normal function and ETD groups. Among the evaluated parameters, the pressure difference between OP1 and CP1 demonstrated the greatest discriminatory performance. A cutoff value of 112.50 daPa yielded a sensitivity of 92.3% and a specificity of 58.7% for differentiating the ETD and normal function groups. However, the modest specificity and the fact that this cutoff was derived and evaluated within the same study cohort should be considered when interpreting its clinical significance. Therefore, the proposed OP1–CP1 cutoff should be regarded as a preliminary exploratory screening threshold rather than an established diagnostic criterion and requires validation in independent populations.

Although a cutoff value of 212.50 daPa yielded a more balanced sensitivity (66.7%) and specificity (65.3%), the 112.50 daPa threshold achieved the highest Youden index and therefore provided the greatest overall discriminatory ability. These findings suggest that the optimal cutoff value may vary depending on the intended clinical application, with the 112.50 daPa threshold favoring overall diagnostic performance and the 212.50 daPa threshold offering a more balanced sensitivity–specificity profile.

Notably, the optimal cutoff value was lower than the mean value observed in the ETD group. This discrepancy is likely attributable to the substantial variability within the ETD group, which has also been reported in previous studies [27]. Because ETD encompasses a broad spectrum of functional impairment, considerable overlap in MIDT measurements between normal function and ETD ears is expected. Consequently, the cutoff value that maximizes diagnostic performance does not necessarily correspond to the mean value of the ETD group. From a clinical perspective, accurately identifying ears in which the Eustachian tube fails to open during the MIDT is more important than reducing false-positive findings, as failure of passive or active tube opening is a key characteristic of ETD. Therefore, minimizing false-negative results is particularly important when evaluating patients with suspected ETD. Accordingly, the cutoff value of 112.50 daPa, which provides higher sensitivity, may offer greater clinical utility for screening and identifying patients with Eustachian tube dysfunction.

Overall, the MIDT findings suggest that patients with ETD require higher pressure to achieve Eustachian tube opening, indicating impaired passive opening compared with normal function ears. Higher residual middle ear pressure after repeated swallowing further reflects impaired active opening and incomplete pressure equalization. Correlation analysis demonstrated significant positive correlations among OP1, CP1, and RP3, whereas all three parameters were negatively correlated with the OP1–CP1 pressure difference. These findings indicate that impaired passive Eustachian tube opening is closely associated with reduced pressure equalization during swallowing, supporting a relationship between passive opening and active ventilatory function. Several limitations of this study should be acknowledged. First, because the MIDT instrument has a maximum measurable pressure of 400 daPa, ears without detectable Eustachian tube opening were assigned a value of 400 daPa for OP1, CP1, and RP3 in the statistical analysis. Consequently, the actual values in these cases may have exceeded the measurement limit or remained unmeasurable, potentially introducing a ceiling effect and influencing the statistical analyses. Second, the proposed OP1–CP1 cutoff value was derived and evaluated using the same study cohort. Therefore, this threshold should be considered a preliminary finding, and further validation in independent cohorts is required before it can be recommended for routine clinical use.

Taken together, MIDT parameters showed good agreement with sonotubometry-based functional classification and may serve as a useful adjunctive tool for evaluating Eustachian tube function [20,27,28,29]. According to the diagnostic criteria proposed by the Japan Otological Society (JOS), the diagnosis of obstructive Eustachian tube dysfunction should be established based on a combination of clinical symptoms, objective middle ear findings, and the results of Eustachian tube function tests. Among the recommended functional tests, sonotubometry is considered a reliable objective method for evaluating active Eustachian tube opening and has therefore been widely used as a reference test in the functional assessment of ETD. For this reason, sonotubometry was adopted as the reference method for classifying the study groups in the present study. Compared with the sonotubometry-based classification, all MIDT parameters demonstrated significant differences between the normal function and ETD groups and showed significant correlations with Eustachian tube opening duration. These findings indicate that the MIDT provides functional information comparable to that obtained by sonotubometry and supports its potential role as an objective method for evaluating both passive and active Eustachian tube function in patients with ETD, particularly in cases where conventional tympanometry-based tests cannot be performed because of tympanic membrane perforation [20].

Patients with ETD exhibited impairment of both passive and active Eustachian tube function, resulting in inadequate middle ear ventilation and incomplete pressure equalization despite Eustachian tube opening. These findings support the clinical value of MIDT, which was comparable to established physiological tests, including the nine-step test, the Williams method, and sonotubometry. Furthermore, the significant correlations between all MIDT parameters and Eustachian tube opening duration measured by sonotubometry suggest that MIDT reflects both passive and active Eustachian tube function, while the OP1–CP1 pressure difference may serve as an indirect indicator of Eustachian tube opening duration.

Therefore, MIDT has the potential to provide a comprehensive assessment of Eustachian tube opening function, particularly in patients with tympanic membrane perforation, for whom conventional physiological tests such as the nine-step test and the Williams method are not applicable. In these patients, MIDT may serve as a valuable alternative for evaluating both passive and active Eustachian tube function.

## 5. Conclusions

In the present study, MIDT was performed following grouping based on sonotubometry findings to evaluate the correlation between the two tests and investigate the characteristics of each MIDT parameter. MIDT parameters demonstrated good discriminatory ability between ears with normal and abnormal sonotubometry findings and may be useful as an adjunctive method for evaluating Eustachian tube function [30,31]. Previous studies have demonstrated a significant association between the Williams method (226 Hz) and sonotubometry [32,33]. Similarly, the present study demonstrated a significant correlation between MIDT parameters and sonotubometry findings. Moreover, unlike several conventional Eustachian tube function tests that primarily evaluate either passive or active Eustachian tube opening, MIDT may provide complementary physiological information by assessing both passive opening induced by pressure loading and active opening during swallowing within a single examination. The present study demonstrated good agreement between sonotubometry and MIDT, suggesting that MIDT may serve as a useful complementary physiological test for assessing Eustachian tube function. However, these findings do not establish MIDT as a definitive diagnostic test for ETD. Accordingly, MIDT may serve as a useful complementary physiological test for evaluating Eustachian tube function in patients with tympanic membrane perforation. However, the proposed OP1–CP1 cutoff value should be interpreted cautiously because it was derived and evaluated in the same study cohort. Further external validation in independent populations and comprehensive clinical assessment are required before this threshold can be recommended for routine clinical use.

This study has several limitations. First, MIDT was evaluated only in the inflation mode with a maximum pressure of 400 daPa; therefore, future studies should investigate higher inflation pressures and deflation-mode MIDT. Second, although the OP1–CP1 cutoff value of 112.5 daPa showed the best diagnostic performance, its modest specificity and the use of sonotubometry as the reference method indicate that further validation in independent cohorts and in combination with other physiological and clinical assessments is required before routine clinical application. Third, because this retrospective study included only adults with chronic tympanic membrane perforation secondary to chronic otitis media, detailed clinical characteristics were not consistently available, and the findings should not be generalized to other populations. Future prospective studies incorporating multiple Eustachian tube function tests and comprehensive clinical evaluation are warranted to further establish the clinical utility of MIDT.

## Figures and Tables

**Table 1:** Differences in age and gender of each group.

		Gender	
	Age (SD)	Men (%)	Women (%)
Normal function	59.85 (12.74)	41 (35.04%)	76 (64.96%)
ETD	56.89 (14.41)	20 (26.32%)	55 (72.37%)
*p*-value	>0.05	>0.05	

ETD, dilatory Eustachian tube dysfunction; SD, standard deviation.

**Table 2:** Differences in MIDT parameters between the groups.

	Normal Function(SD)	ETD (SD)	*p*-Value
OP1	344.78 (58.03) daPa	380.15 (34.63) daPa	<0.001
CP1	107.85 (94.24) daPa	258.40 (152.82) daPa	<0.001
OP1-CP1	236.93 (87.71) daPa	121.75 (133.61) daPa	<0.001
RP3	58.73 (82.33) daPa	237.19 (162.75) daPa	<0.001

CP1, first closing pressure; ETD, dilatory Eustachian tube dysfunction; OP1, first opening pressure; RP3, residual pressure after three openings; SD, standard deviation.

**Table 3:** Difference in the “Not detected” rate between the groups.

	Normal Function (SD)	ETD (SD)	*p*-Value
Not detected	4 (3.4%)	35 (46.7%)	<0.001

ETD, dilatory Eustachian tube dysfunction.

**Table 4:** Correlation between MIDT measurement parameters and duration of Eustachian tube opening.

	OP1	CP1	OP1-CP1	RP3	Eustachian Tube Opening Duration
OP1		0.725 *	−0.367 *	0.600 *	−0.445 *
CP1	0.725 *		−0.809 *	0.775 *	−0.521 *
OP1-CP1	−0.367 *	−0.809 *		−0.679 *	0.430 *
RP3	0.600 *	0.775 *	−0.679 *		−0.602 *
Eustachian tube opening duration	−0.445 *	−0.521 *	0.430 *	−0.602 *	

* *p* < 0.001. CP1, first closing pressure; OP1, first opening pressure; RP3, residual pressure after three openings.

**Table 5:** Cut-off value for pressure change when opening the Eustachian tube.

OP1-CP1	Sensitivity	Specificity	Sensitivity + Specificity
103.00	0.932	0.547	1.478
106.00	0.923	0.547	1.470
108.50	0.923	0.560	1.483
112.50	0.923	0.587	1.510
129.00	0.897	0.587	1.493
138.00	0.880	0.587	1.476
144.50	0.872	0.587	1.458
150.50	0.872	0.613	1.485
156.50	0.383	0.613	1.451
163.50	0.821	0.373	1.447
173.00	0.795	0.627	1.413
185.50	0.761	0.640	1.401
191.50	0.752	0.640	1.392
198.00	0.744	0.653	1.397
204.50	0.718	0.653	1.371
209.00	0.692	0.653	1.346
212.50	0.667	0.653	1.320
218.00	0.650	0.667	1.316
224.00	0.615	0.667	1.282
229.50	0.590	0.320	1.270
234.50	0.573	0.680	1.253

## Data Availability

The data presented in this study are not publicly available due to privacy and ethical restrictions.

## Abbreviations

The following abbreviations are used in this manuscript:

PET	patulous Eustachian tube
ETD	Eustachian tube dysfunction
MIDT	modified inflation–deflation test
TTAG	tubo-tympano aerodynamic graphy
OP1	First opening pressure
CP1	First closing pressure
RP3	Residual pressure after three openings
